# Extracts and Gleanings

**Published:** 1876-08

**Authors:** 


### Extracts and Gleanings.

DR. WILLARD PARKER, of New York, at the close of an article on cancers, sums up as follows: With regard to treatment I have not much to say. The methods employed may be embraced under the following heads:

1. Amputation; 2. Caustic applications; 3. Compression; 4. Electrolysis; 5. Medication; 6. Moral treatment.

In the superficial cancer of the breast it is very well to use caustics. The same thing may be said with regard to cancers upon the face. The treatment with caustics in that region is good surgery. When the tumor is situated to any extent below the surface, the idea of caustics is bad surgery.

In two cases which have come under my observation, one died within four days, poisoned by the material used for the caustic application, and the other never reached her home alive.

With regard to the treatment by the use of compression, compressed sponges being usually employed, I have seen no good from it.

With regard to electrolysis, I have seen nothing in it yet to give me any confidence whatever in its use. I have nothing, however, to say against its use. There may be something of value in it, and it should be thoroughly tried. The day was when no knowledge was had with regard to a successfull method for the treatment of syphilis, but now we know that by the proper use of proper remedies, that disease can be cured, and charlatanism has left that field almost entirely.

With regard to the internal treatment of cancer, I believe very much in it.

I believe that the day must come when something will be accomplished by the aid of internal remedies. Of the remedies now used, arsenic is perhaps the one which commands my confidence more than any other. There is another point in the treatment of cancer which I conceive to be of great importance, and that is the moral condition of the patient. I believe that it is impossible to cure our patients of cancer unless they are buoyed up by hope. Their surroundings should be of a character that will give them the greatest possible amount of comfort and happiness. Keep them in the sun-light of enjoyment, for darkness is the soil in which cancer flourishes.

Now we come to consider an important question: Do we accomplish any good by operations?

There are some who say, never operate. I think this opinion comes from the older members of the profession, who are inclined to look beyond the simple performance of the operation. The younger men, many of them, say operate. Upon this question I perhaps can do no better than to refer you to the opinions of two men who are among the most experienced of the profession, and who have had abundant facilities for making observations. I refer to Paget and Sibley, both of London.

Mr. Paget has shown, from his statistics, that the average length of life after operation is forty-three months, and that average length of life without an operation is fifty-five months. Mr. Sibley has shown from his statistics that the average length of life after an operation is fifty-three months, and that the average length of life without an operation is thirty-two and one-half months. Here are the results of observations made by two distinguished authorities. I think all that I am justified in saying upon this point is, that every case must be taken by itself, looked at with all its surroundings, before a decision is given for or against an operation.

The dangers in an operation are not great, if it is decided to perform it.

The following may be regarded as the indications, when attempting to decide upon any given case:

The older the patient, other things being equal, the more favorable for operation. If the cancer has extended so that we have secondary cancer, it is not surgical to operate. Therefore, when the axillary glands are involved, or when the skin is involved, and we have the local and constitutional disease both existing, I regard it as unfavorable for an operation. When the tumor is isolated, and there are no secondary manifestations, the conditions are favorable for on operation, and the sooner it is performed the better is the chance of preserving the life of the patient.

If a patient comes complaining of an irritable tumor of the breast, apparently connected with some disorder of menstruation, I should recommend, first, careful attention to the general health; and second, if found increasing in size, to remove it at once. There is another condition in which I would operate, and that is in the sloughing cases. Then it is done simply to make the patient more comfortable. Practically speaking, these cases do not belong to secondary cancer, and the operations are not unfavorable. But with all these cases we must use our own discretion. Select the cases, and give the benefits and advantages of an operation.

Now a few words with regard to the hereditary character of cancer. In the cases which are found in my tables, the cancer taint was present in only 28 of the whole number, 236, whose history upon this point was obtained.

Within the last year I have been examining the Registrar's Bureau of Statistics in this city, and I find, in a period of time extending over about 70 weeks, there were only 532 deaths from cancer of all kinds and in all organs, while from pulmonary consumption alone there were 6,219 deaths, or as 1 to 11½.

When compared with Bright's disease it is found that about three times as many die from that disease as from cancer. From the statistics of the Registrar's office for the last five years, the following proportion of Americans and foreigners who died of cancers are found:

Americans, 68; foreigners, 154; negroes, 5. Savages rarely have the disease.

It would seem as if this disease of the breast is found in certain conditions of life, and that in these conditions it is upon the increase.

Without pursuing the discussion of this subject farther, I will close by saying that the conclusions to which I have arrived are chiefly as follows:

1. That the disease is not hereditary, or if so, in a very limited degree.

2. That the disease begins as a local disease positively and purely. It becomes constitutional, just as syphilis begins a local disease and becomes constitutional.

3. That the disease occurs in those of vigorous health, instead of being connected with those conditions in which consumption occurs.

4. That cancerous parents may beget tuberculous offspring. That is, feeble constitutions arising from the effects of cancer will not beget cancer, but the diseases which follow in their line are tuberculous.

5. That the moral condition has a powerful influence on the development or the prevention of the development of cancer.

6. I am very forcibly struck by the parallelism and analogy existing between cancer and syphilis. Both begin by local irritation. Syphilis is innoculable, but cancer has not been proven so to be. In this respect they differ from each other. We have secondary syphilis, and we have secondary cancer. We have tertiary syphilis, but perhaps it cannot be said that we have tertiary cancer, unless it can be said that cancer is tertiary when it affects the bones, as it sometimes does.

In conclusion, I have to say, that we must not give this subject over as an unprofitable one for study and observation.

Many diseases have run rampant which finally have been made to yield to treatment, and we may hope that the same thing may yet be accomplished with reference to cancer. The work of the histologist and pathologist may yet bring us into the light, and the day may come when we can say of cancer as we can say of syphilis, it can be cured.—*Medical Record.—Cincinnati Medical News.*

*Digitalis in Acute Diseases.*—Dr. James Little, in a paper read at a meeting of the College of Physicians of Indiana, says:

"Under circumstances such as these the author had employed digitalis in more than twenty cases, including six of typhus, one of rheumatic fever, and the remainder of enteric fever. The preparation used was the tincture, given in half-drachm doses every three or four hours, and rarely every hour. The administration of the remedy was discontinued after the pulse had fallen to 80, and, except in one case, the action of the drug was supplemented by wine or brandy, given in cordial or stomachic doses. In one case of rheumatic fever, digitalis was used alone. The patient, a merchant, aged 35, had symptoms of a rheumatic attack towards the close of last October. Six months previously he had suffered from severe dyspepsia, with much cerebral disturbance. On October 26th he remained in bed, the heart was very weak, and the temperature was 102°. Tincture of the perchloride of iron was given in twenty-minim doses every fourth hour. Four days later, signs of commencing cardiac complication appeared. The evening temperature on November 6th was 103.6°, the highest during his illness. On November 11th he was delirious at night, and on November 18th he had been one hundred hours without sleep, his pulse was feeble (100 per minute), and the first sound of the heart was absent. As stimulants could not be borne, half-drachm doses of tincture of digitalis were given every hour. After eight doses, the patient fell asleep. Nausea having afterwards set in, the tincture was withheld, and one-eighteenth of a grain of atropia, one-fortieth of a grain of digitaline, and a fourth of a grain of morphia were injected hypodermically. The patient did well."

The Chairman expressed his deep sense of the value of Dr. Little's communication, and alluded to the novelty of the application of digitalis in functional affections of the heart. Dr. Hayden could not but look upon digitalis as a cardiac tonic, the "opium of the heart," as it had been termed. He recalled the practice of Mr. Jones, of Jersey, who used the drug freely in delirium tremens. Dr. Hayden generally gave ten minims of the tincture, in combination with perchloride of iron and spirit of chloroform. He believed that digitalis was useful only when it acted on the kidneys. It was of great advantage in fatty heart with dilatation of the cardiac chambers. Dr. Grimshaw had used digitalis six years ago in a case of acute rheumatism with nervous symptoms, similar to the one described by Dr. Little. The patient was delirious except when under the influence of full doses of digitalis (given twice or thrice a day). The heart was very weak. In a subsequent attack digitalis failed, while brandy succeeded; the disease, however, assumed the character of pyæmic rheumatism, and the patient died. He believed the infusion to be the most reliable preparation of the drug. Large doses were especially dangerous in delirium tremens, and in all instances caution was necessary. He had used strychnia with much success as a cardiac tonic in fever. Dr. H. Kennedy relied most on powdered digitalis. The drug had long since been employed in maniacal cases. He considered that, in order to test its efficacy in a satisfactory way, the remedy should be given alone. Vomiting was a dangerous symptom. Dr. W. G. Smith dwelt on the importance of the class of cardiac remedies, and remarked on the inutility of experiments on the lower animals apart from clinical observation and experiments. Digitalis was proved to be a direct cardiac stimulant. The question of tolerance of the drug turned on the value of the preparation employed. The active principle, digitaline, had recently been isolated in France as a crystalline substance, very unlike the amorphous powder at present in use, and which was of most uncertain strength.

*Neurosis of the Joints and Their Treatment.*—The following summary of the subject is from a paper by Moritz Meyer, of Berlin. Cases of painful affections of the joints, as described by Sir Benj. Brodie, some fifty years ago, under the name of "Hysterical Affections of the Joints," and which often simulate inflammation of those parts, have recently received attention from Stromeyer and Esmarch. The seat of the disease is in the small branches of sensitive nerves supplied to the capsule of the joints, and the integuments in the neighborhood. It is marked by pain, often of such a degree as to prevent the use of the joint, and to result in its fixation in a certain position, being often the only cause of an immobility which lasts for years or for life. According to Berger, it may originate in nervous subjects without any direct cause, while in healthy persons it is often the result of mental or emotional causes, of trivial or more serious injuries of the joints, or of irritations reflected from the gastric, urinary, or genital organs. Its discrimination from an inflammatory affection, especially when it is ascribed to a traumatic cause, is of great importance, particularly in reference to the treatment applicable to one or the other case. The following points will aid in differentiating it from affections which it resembles: 1. Nocturnal pain is commonly absent. 2. The joint will be found more sensitive to slight movements and handling than to rougher manipulation. 3. Œdema and swelling of the skin and cellular tissue are often observed to appear and then vanish. 4. There are sometimes periodical changes in the temperature of the affected joint. 5. The muscles are relatively but little shrunken in spite of long periods of absolute rest. 6. There is great want of agreement between the trivial objective symptoms and the severe complaints of the patient. 7. It is often possible to bend the joint without complaint of pain, when the patient's attention is distracted. A spontaneous cure is not unfrequently wrought by a variety of causes working through the mind or passions, and where the affection is of reflex origin, by the cure of the primary disease. Stromeyer and Esmarch have attained the best results by a general tonic management, supplemented by the kneading and passive motion of the affected joints, and they specially emphasize *the active use of the limb.* To these remedial measures Meyer adds the recommendation of a strong induced electrical current, to be applied directly to the painful joint, for which he claims anæsthetic and anodyne effects, as well as an excellent sedative effect upon the mind of the patient. Meyer appends an account of four cases observed by himself in which the results of faradization are well illustrated, and also one of Esmarch's, where he and Stromeyer proved most strikingly the efficacy of the treatment by active exercise.—*Sitzungsbericht der Berliner med. Gesellsch.*, 1874.—*Allg. med. Central-Ztg.*

*Emulsions of Cod Liver Oil.*—Wm. G. Moffit (*Am. Jour. of Pharmacy*) gives the following formula. He says:

From some experiments tried I would select the following formula for an emulsion of cod-liver oil. This is generally found to give satisfaction, and to remain unaltered for some time:

COD-LITER OIL MIXTURE.

| | | |
|---|---|---|
| R. | Pulv. Acaciæ, | oz. ii, |
| | Sacch. Alb., | oz. i, |
| | Aquæ, | oz. iv, |
| | Spts. Vini Gall., | oz. iv, |
| | Syr. Rubi Idæi, | oz. i, |
| | Ol. Gaultheriæ, | gtt. xviii, |
| | Ol. Morrhuæ, | oz. viii. |

M. ft. emuls.

This contains about 50 per cent. (42 per cent, by measure, Edit. *A. Jour. Ph.*) of oil, and is a very pleasant preparation.

In the above recipe there will be found by some an objection in the use of the brandy, on account of the alcohol which it contains being capable of precipitating the gum from solution. This can be obviated in a great measure by adding it last. It has, however, the property of preserving it for a length of time.

An emulsion is by far the best method of incorporating cod-liver oil with other medicines. Iron is often introduced. This is easily done by adding a soluble salt to the mixture. In the following formula a concentrated solution of pyrophosphate of iron is used, which keeps well, and is a very useful addition to our list of cod-liver oil mixtures. It is, I think, one of the easiet and most convenient ways of administering iron in combination with cod-liver oil, and is much liked by those who in their practice have had occasion to use it:

COD-LIVER OIL IN COMBINATION WITH IRON.

R. Pulv. Acaciæ,........................oz. i,
Pulv. Sacch. Alb.,..........................oz. ss,
Aquæ,........................................oz. iv,
Alcohol, ........................................oz. i,
Ol. Morrhuæ,..................................oz. v,
Sol. Ferri Pyrophosph.,....................gtt. cc,
Ol. Amygdal. Amar.,........................gtt. v.

M. ft. emuls.

A new preparation of cod-liver oil has recently come under the attention of physicians and pharmacists, namely, that of lacto phosphate of lime and cod-liver oil. The most advantageous manner of preparing this is a matter of dispute. I have found the following to answer all the purposes indicated:

LACTO-PHOSPHATE OF LIME AND COD-LIVER OIL.

R. Pulv. Acaciæ,..............................oz. i,
Pulv. Sacch. Alb.,..........................oz. ss,
Liquor Calcis,................................oz. iii,
Alcohol,........................................oz. i,
Ol. Morrhuæ,..................................oz. i,
Sol. Calcii Lacto-phosph,................q. s.
Ol. Gaultheria, ..............................gtt. v. M.

In the above, lime-water is substituted for water, to neutralize any excess of lactic acid in the solution of lacto-phosphate of lime. This often gelatinizes, on account of the sucrose being converted by the action of lactic acid into glucose, and thus rendering it so thick and ropy as to be unfit for use.

The solution of the lacto-phosphate of lime is made by dissolving phosphate of lime in lactic acid. The solution should be assayed, and water added to make the required amount, which is two grains to the teaspoonful of mixture. The solution should be filtered in order that the emulsion should be perfectly white.

The phosphate of lime and cod-liver oil is also a new preparation, and is sometimes used in preference to the above. It is made by adding freshly prepared phosphate of lime to the emulsion, and stirring constantly until it is thoroughly and uniformly mixed. On standing, it, however, lets fall the phosphate of lime, and requires to be well shaken before it can be used.

In all the above preparations much labor can be avoided by the use of a patent churn, which may be had to hold thirty or forty gallons of the mixture. In the process of making an emulsion of this kind on a large scale, care should be taken that the mucilage is perfectly uniform and free from lumps before the oil is added.

*The Treatment of Diarrhœa in Children.*—In a recent (July 8) number of the Boston *Medical and Surgical Journal*, Dr. J. P. Oliver presents some practical hints concerning the treatment of that common form of diarrhœa in children which depends upon the non-digestion of the food. The disease is most apt to attack children between the ages of six months and two years, and during the hot season of the year. The attack is often excited by taking some indigestible article of food, or after an exposure to cold a diarrhœa is produced, which tends to become chronic. The stools are acid, and consist of a peculiar, light-colored, pasty-looking substance, mingled with mucus, and perhaps streaked with blood. Progressive emaciation and debility ensue, and, finally, a fatal termination either in connection with cerebral symptoms or as a result of some intercurrent affection. The main points in Dr. Oliver's treatment of this form of diarrhœa appear to have reference to deficiency in the catalytic force displayed in the process of digestion and to the excessive acidification with which the digestion is associated. When undigested food is found in the dejections, it is inferred that the child's diet is not adapted to its digestive powers. If the diet has consisted of pure milk, the addition of gelatine, to the milk as recommended by Meigs and Wyman, will often assist digestion indirectly by preventing coagulation in the stomach, and thus enabling the "digestive agents" to operate at a greater advantage. As a rule, a farinaceous diet is unsuited to infancy, on account of the inability at this early period of life to affect the transformation of starch into sugar. In the so-called Liebig's soup, however, the presence of diastase in the malt supplies the defective ferment in the stomach, and assists to digest the flour which the soup contains. Sometimes this preparation, therefore, is well borne in quite young children. Its effect must be tested by inspection of the stools. For children who can be supplied with cream or good milk, no other food is thought necessary. The excessive acidification is best combated, according to Dr. Oliver, by means of the bicarbonate of potassa, to which the addition of an aromatic is advised. The potash is preferred from the fact of its being a natural constituent to the milk. When the powers of life begin to fail, and but a small quantity of food can be taken at the time, beef-tea is given, to which a little saccharated pepsin is added. If the fontanelle becomes much depressed, stimulants are indicated.

*The Unmanageable Vomitings of Pregnancy.*—The *Journal de Medicene et de Chirurgie* quotes a communication from M. Tarnier respecting a case in which, in a multipara in the third month of pregnancy, serious unmanageable vomitings were arrested by the simple application of a plug of wadding to the vagina. He collates with this fact three cases published in the *British Medical Journal*, in which Dr. E. Copeman saw very severe vomitings arrested by the dilitation of the neck of the uterus. In the first case, the digital dilatation of the neck was to have been followed by puncture of the membranes to induce abortion at six months. A fortunate delay demonstated to Dr. Copeland that dilatation had a great influence on the arrest of the vomitings. Pregnancy went on in due course, and the patient was delivered at the proper time. In a second case, the result was intentionally sought and obtained in the second month of pregnancy. In a third, pregnancy had reached the eighth month. The vomitings were also stopped, and the patient was delivered eighteen days afterwards, when she had already regained some strength. The plug and the dilatation of the neck are two mechanical methods which, in the opinion of the writer who comments on these cases in the *Lyon Medical*, are very rational, though undoubtedly acting by a different mechanism. The plug prevents the shaking about of the womb; the dilatation of the neck detaches the membranes over a certain space, and prevents the twitching or distension of the internal orifice.—*British Medical Journal.*

*Hemiplegia; Use of Strychnia.*—A patient, aged twenty-five, entered the hospital suffering from right hemiplegia. The history pointed to its gradual invasion, and the only cause which could be assigned was an injury of the head, received when he was a child. Strychnia in the following form was administered:

| | | |
|---|---|---|
| R. | Strychnia | gr. j. |
| | Acid. acetic | dr. ss. |
| | Tr. card. co | dr. ss. |
| | Spts. vin. rect | oz. jss. |
| | Aquæ | oz. jss. |
| | Syrup. simp. ad | oz. jv. |

Teaspoonful three times a day. Under this treatment the case is improving.—*N. Y. Med. Jour.*

*Sore Nipples.*—I have had a large obstetric practice as an English physician, and have never had a bad case of sore nipples. For many years, when the nipples became slightly sore, I at once applied zinc shields; but of late years, instead of allowing the zinc to combine with the lactic acid of the milk, I have applied a preparation of sulphate of zinc and lactic acid (in fact, lactate of zinc) and glycerine with starch, between the times of sucking. I think if you try this you will find it unfailing, and not only a "prophylactic," but a specific in the true sense of the term.—FLEISCHMANN.—*The Clinic.*

*Rheumatism.*—Dr. William Corson has derived much success in this disease with the following:

| | | | |
|---|---|---|---|
| R. | Phosphate of ammonia | dr. iss. | |
| | Tincture of colchicum seeds | oz. j. | |
| | Tincture aconite root | dr. ij. | |
| | Simple syrup | oz. iij. | M. |

Teaspoonful every three or four hours.—*Philadelphia Med. & Sur. Reporter.*

*Diphtheria.*—Dr. Benson (*Canada Lancet*) says: When called to a case of diphtheria, I first of all clear out the bowels by a dose calomel, followed in a few hours by a little senna or Rochelle salts. If there is much heat of skin, as will be shown by the thermometer, a warm bath will give very great relief, and after the bowels have been freely acted upon I commence with the chlorine water. To patients from 10 to 12 years and upwards I give one tablespoonful; from 6 to 12, one desert spoonful. and under 6, one teaspoonful every three hours. In all cases it should be given without any addition of water. A towel saturated with cold spring water is to be applied to the throat and neck, the cold renewed as the towel becomes warm. The patient is to have small pieces of ice on a plate by the bedside, a piece of which is to be kept constantly in the mouth. The diet should be nutritive; beef-tea, tea, milk, eggs, and such like; but no stimulant is required, unless the prostration should be alarming. In some cases which are mild and occur in young children, a mixture containing tincture of iron, chlorate of potash, and dilute hydrochloric acid, may suffice to cure, but in no case can the same dependence be placed in it as chlorine water.

*Radical Treatment of Prostatic Hypertrophy.*—Prof. Heine (*Langenbecks Archiv*) has cured six cases of prostatic hypertrophy with iodide-injections, and now recommends the parenchymatous injection of moderately concentrated solutions of iodide of potassium; he states that the operation is not severe, and can be borne by old and weak individuals, because the diminution of the hypertrophied organ takes place without suppuration. When its volume is diminished, the secondary affections of the bladder are also relieved, provided they have not attained a high degree. The operation is performed by placing the patient on his side at the edge of the bed, and introducing the oiled index-finger of the left hand into the rectum to the point where it is intended to make the injection. An exploring trocar is then introduced on the finger, the stilet having been withdrawn into the canula, and the puncture is made. The stilet is then withdrawn from the canula, which is filled with the solution in a syringe. When the canula has been filled, an air-tight syringe is attached to the canula and the injection performed. The median line of the prostate should not be chosen, as a small artery takes its course in this location. The author's solution is: Iodidi potass. dr. ij, tr. iodinii oz. ij, aq. destil. oz. ij.—*Med. Chir. Centralblatt*, 31, 1874.

*Ergot in the Treatment of Galactorrhœa and Mastitis.*—Dr. Schtscherbinenkoff observed during an epidemic of raphania that, associated with the symptoms of ergotism, the secretion of milk in nursing women was often diminished or arrested. Recognizing the fact that an accumulation of milk in the ducts is the great cause of perenchymatous mastitis, he prescribed the ergot of rye for two women, who in former confinements had suffered from this trouble, giving it as soon as there was swelling of the glands. The results were most favorable. In so-called milk fever he also gave ergot, combined with quinine, in doses of grs. v.–x. of each three times a day. He also found it useful in arresting the secretion of milk after weaning the child. He gave as much as a drachm daily for a week, without unfavorable results.—*Sitzungsberichte d. Ges. russ. Aerzte in Moskau*, 1874. –*Allg. Med. Cent. Zeit.*, May 19, 1875.

*Calculus of the Bronchus.*—M. Burdel reported the following remarkable case to the Paris Academy of Medicine:

A woman, aged fifty-seven, was suddenly seized, without any known reason, with a severe chill, followed by fever. This recurred on the second day, and soon took a tertian type, for which quinine was given with apparent effect, checking the fever for several days. However, there was a reappearance in a few days; the slight cough the patient had complained of became worse, and at times there was a sharp pain in the right chest on coughing. No abnormal physical signs could be obtained on auscultation or percussion. This condition continued for four weeks; the access of fever appeared very irregularly, at times being very sharp, and occasioning alarm. Finally, during an attack of coughing, the patient expectorated a hard mass eleven millimetres long, irregular in shape, of the thickness of a goose-quill, and forked at one extremity. Immediate relief followed the expulsion of this concretion. The cough and fever disappeared as if by magic.—*Gaz. Hebdom,* April 28, 1876.

*Local Application of Creosote in Hæmorrhages.*—Hæmonrhages from the nasal, pharyngeal, and oral cavities often lead to death, because of the inefficiency of the remedies used to check them. The sanitary physician, Dr. Bottger, of Dessau, finds creosote to be far superior to all other styptics for this purpose. In the case of dental hæmorrhages, a small tampon, impregnated with a thick mixture of creosote in substance and alumen kinosatum is to be pressed into the bleeding alveolar cavity. If the hæmorrhage does not stop at once, another similar compress should be superimposed, and the pressure increased with the finger. In obstinate nasal hæmorrhages, plugging the nose with charpie tampons, impregnated with the same mixture, is uniformly successful. The author gives the histories of a number of cases which prove creosote to be an extremely valuable local styptic. —*Memorabilien*, xx. Jahrgang, 11. Heft.

*Diptheriain the Adult Tracheotomy.*—A man was taken into hospital, suffering from diptheria. He appeared to be moribund, and shortly after admission respiration nearly ceased. It was considered advisable, however, as a last resort, to practice tracheotomy. This operation was exceedingly happy in its results, inasmuch as the patient returned to consciousness, and was completely relieved of his dyspnœa.

He said that he felt quite comfortable, and was very thankful that the operation had been performed. About six hours afterward he sank and died of exhaustion. There was no return oı dyspnœa. At the optopsy false membrane was found at the base of the tongue and in the larynx. There were also evidences of phthisis and pleurisy.—*Roosevelt Hospital*, *New York Medical Journal.*

*Myelitis a Sequel of Diarrhœa.*—A woman suffering a severe attack of diarrhœa about a year ago, and shortly after recovering from it noticed a sensation of weakness in her limbs. There was no pain, however, nor the sensation of any constricting band around the body.

The paralysis increased, but only a short time before death did she complain of any anæsthesia. At the autopsy the entire cord in the dorsal region was found to be softened.—*Roosvelt Hospital, New York Medical Journal.*

*Electrolysis in Nævi.*—Mr. Knott, Medical Superintendent of Galvanism to St. Mary's Hospital, London, has reported forty cases of nævi successfully treated by the above method. He uses Stohrer's and Myer and Meltzer's continuous batteries, and judges according to the size of the nævus how many cells to use; six or eight is about the average if the battery be in good order.

He says that if the nævus be small, he uses one or two needles attached to the negative pole, and one to the positive, and passes them into the tumor; but if it be large, he puts on several needles in the negative cord, and uses a charcoal point with the positive. After the needles have been in the tumor a short time decomposition begins to take place; this is shown by bubbles of gas passing by the side of the needles. A clot is then formed, the tumor turns to a bluish white, and in this clot fibrous degeneration takes place, and an ultimate cure is the result. The advantages of the galvanism are its certainty of action, its safety, the faintness of the cicatrix, and the cessation of pain directly the operation is over. He has used every other method, and thinks this by far the best.—*The Lancet,* March 20, 1875.

*Liquor Opii Sedativus.*—(Battley's Sedative Solution.)

Hard extract of opium..........................3 oz.
Boiling water......................................30 oz.
Alcohol ..............................................6 oz.
Water, sufficient to complete..................40 oz.

Dissolve the extract in the boiling water, filter when cold, and add the other ingredients. Dose, ten to thirty drops. Battley's solution is said to be less narcotical than opium or common laudanum. Dr. Christison says that twenty drops of Battley's solution are equal to thirty drops of the officinal tincture.

*Leptandra as a Purgatave.*—Dr. Wollen writes to the Louisville *Med. News:* "During the past few years I have been prescribing the *fluid extract of Leptandra*, with results so gratifying that I desire to call attention to the virtues of the above-named drug. Whether or not it has any specific action upon the liver I cannot say, but I am certain that it is one of the most efficient and safe purgatives that we have, and can be used without fear of debilitating the patient; in this respect widely differing from podophyllin, a remedy so often prescribed in hepatic disorders.

"In that class of cases usually called bilious, whatever that may mean, where there is loss of appetite, with a heavily-coated tongue and constipation of the bowels, I know of no single remedy universally applicable and useful as the leptandra. It is often well to combine it with the fluid extracts of senna and rhubarb; but whether so combined or given alone, it is certain in its results, and produces purgation without pain, griping or debility. In those cases of obstinate constipation occurring in young infants, where it is necessary to resort to a purgative, it will be found that a few drops of the leptandra added to the syrups of senna and rhubarb will produce the best results.

"It must be borne in mind, however, that the proper dose will vary with the individual. Owing to idiosyncrasy or other cause some patients will need but ten minims at a dose, while others will require as much as a drachm to produce any decided effect. The disagreeable taste of the drug is the only bar to its use, and this may be overcome to some extent by combining it with some syrup that is agreeable to the taste of the patient."—*Med. Brief.*

*Dangers of Absorption of Carbolic Acid.*—The carelessness with which non-professional journals take up certain popular receipts is not always without its dangers, and especially since these often come into the hands of children. For instance, there appeared lately in one of the public prints an article upon the poison of vipers, which recommended that carbolic acid should immediately be introduced within the wound, the acid to be mixed with alcohol *in the proportion of two to one.* Observe the off-hand manner in which a toxic agent is spoken of, as if it were the most inoffensive thing in the world. In order to try the experiment, a cat was selected upon whose skin, denuded of hair alone, a saturated solution of carbolic acid in alchohol, with an equal quantity of water, was rubbed. This produced no effect; but when the same solution was rubbed into a scratch upon the nose two or three times, the animal immediately fell into convulsions, and very shortly succumbed. Prussic acid could not have acted more promptly. The moral of this experiment is obvious.—*Med. Times.*

*On Bromide of Iron and its Preparation.*—To prepare bromide of iron M. Prinie gives the following formula:

| | |
|---|---|
| Iron filings.................................... | 10 parts. |
| Distilled water........ ....................... | 80 " |
| Bromine ............................................ | 21 " |

The iron filings and water are put into a flask, a small quantity is added, and the flask closed with a plug of cotton, to avoid the loss of bromine. It is shaken, and when the vapor of bromine has nearly disappeared, a fresh quantity is added. This process is repeated till the whole of the bromine is used. When the combination is finished the whole is poured—including the excess of iron—into a stopper flask. The solution contains one-third of the weight of bromide of iron. With this normal solution pills and syrup are prepared.

PILLS OF BROMIDE OF IRON.

| | |
|---|---|
| Filtered normal solution............... | 12.00 grams |
| Iron filings.... ... ......................... | 0.10 " |
| Powdered gum arabic................... | q. s. " |
| Powdered licorice......................... | q. s. " |

The solution and the iron are put into a porcelain capsule, and quickly evaporated until the liquid has lost two-thirds of its weight; while still hot it is poured into a porcelain mortar, dry and slightly warm. The two powders are mixed, and added in sufficient quantity to form a consistent pill mass, which is divided into 80 pills, which must be kept in a dry bottle. Each pill contains 0.05 gr. of bromide of iron.

SYRUP OF BROMIDE OF IRON.

| | |
|---|---|
| Filtered normal solution............ | 12 grammes. |
| Gum syrup of orange flower......... | 620 " |

Mix. 31 grammes of this syrup contain 20 centigrammes of bromide of iron.—*Jour. de Pharm. et de Chimie.*

*Mania-a-potu.*—Dr. L. A. Dugas, in a paper read before the Medical Association of Georgia, relates his treatment of this disease. It consists of the administration, once or twice a day, of cold water affusions, of such food as may be relished, and at times of two or three small glasses of toddy. If the patient is very unruly, one or two intelligent persons should attend him day and night, as long as it may be necessary. For the affusions, the patient should be stripped of all garments and seated in a tub, the physician should pour one pitcherful after another over the head in a regular stream, which will pass down over the entire body. The affusion is to be continued from one to three minutes by the watch. The pulse at first full and strong soon becomes small and frequent. The physician should watch this carefully, and cease whenever the patient is entirely rational and the pulse sufficiently subdued. After the patient is rubbed and dried, and the room darkened, he will generally sleep six or eight hours, and awake perfectly rational. With the exception of toddy, all narcotics are eschewed in the management of the case.

*Hot-Water Douche in Neuritis.*—Dr. Gibney, (Louisville *Medical News*) having expanted the usual methods of treatment in nuritis supervening upon neuralgia, says:

Finally, on May 20th, I directed the wife to pour from a pitcher, a distance of three or four feet elevation, water as hot as could be borne over the shoulder, allowing the stream to run down the arm. At the end of a week he returned greatly relieved and enthusiastic on the subject of hot water. The treatment was continued a few wee s longer, and on the 6th of July, 1875, I discharged him cured. No pain whatever existed, and, most remarkable, the spasm was entirely relieved; the normal power had returned to both extensors and flexors; in fact, the cure seemed complete. I had him call October 18th and there had been no relapse. April 19, 1876, I saw him again, and a careful examination of the arm failed to detect any disease or any signs of pre-existing disease. He has not had a twinge of pain for eight months.

*Treatment of Severe Sprains.*—Mr. Sampson Gamgee gives a case of sprain of the ankle treated by compression, with very satisfactory results, and says: "Severe sprains are often serious fractures, though no bone be broken, or only a bit may be chipped off; the ligaments and fasciæ are ruptured, blood being extravasated into the joints, into the sheaths of tendons, and for some distance not infrequently between the layers of muscles. The swelling is great, the pain intense. The orthodox treatment by leeches and fomentations is valueless, compared with circular compression and perfect immobilization. Not only can the patient bear well-applied pressure from the first, however great the swelling and acute the pain, but it may be laid down as a general proposition, to which I have never seen an exception, that in severe sprains effusion is most surely checked, and, once it has occurred, its absorption is most rapidly promoted, while pain is most effectually relieved, by pressure and immobilization. It is as true now as when Velpeau taught it, that 'compression is the sovereign resolvent in contusions with infiltration and swelling.'"—*Lancet*, April 29, 1876.

*Guaiacum in Acute Diseases of the Throat.*—Dr. Fritzinger, of Ashland, Pa., in the *Medical and Surgical Reporter*, extols guaiacum in the treatment of acute tonsillitis and other inflammatory affections of the throat. He thinks it acts first as a local astringent, second as a diaphoretic, third, a laxative, and fourth, a specific resolvent on the inflamed tonsil glands. He prefers the following formula:

R. Potasse chloratis............................dr. j.
Spt. Eth. Nit.......................................dr. iv.
Tinct. Guaiaci.....................................oz. iss.

M. Sig. a teaspoonful every three hours in sweetened water.

It is an old practice revived, with the exception that the powder was formerly used instead of the tincture.

*Mammitts and Mammary Abscess Treated by Bandaging.*—Dr. L. A. Dugas read a paper before the Medical Association of Georgia upon this mode of treatment. To lessen the determination of blood to the mammæ while inflamed, the child is to be suckled as seldom as possible, and general compression sufficient to force out the blood from the distended vessels, and cool applications to the organ are the proper remedies. He has devised a bandage which, he believes, combines all the requisites. It consists of cotton or linen shirting, about ten inches wide, and long enough to surrond the thorax, and to be secured in front by digitations similar to other "many-tailed" bandages. This is to be applied from the axilla down, pass around the chest, and over the mamma and tied in front of the sternum. It effectually compresses both organs, and can be loosened or tightened without any difficulty. The child may be nursed through an aperture for the nipple made in the bandage. As soon as the congestion becomes painful, or threatening, the bandage should be applied with such moderate tightness as will relieve pain, and the treatment should be continued until the trouble has been entirely subsided. When supperation takes place, the pus should be let out by free use of the bistoury, and a linen tent renewed daily. There should also be an opening in the bandage opposite the incision for the escape of pus. This bandage is adapted to every stage of mammary inflammation. If too late to prevent suppuration, it will lessen the size of the abscess, and after an opening has been made for the escape of pus, it will bring together the walls of the abscess and hasten the cure. One of its greatest advantages is that it relieves the patient of all pain as soon as it is applied, by removing the distension to which the unsupported tissues are subjected by the congestion, or the accumulation of pus.

"*No More Ovariotomy.*"—Under the above startling title, says the London *Medical Times and Gazette*, we find a note in the *Surgical Centralblatt* for February 12, taken from the *Wiener Med. Presse*, 1875, No. 52, by Dr. Semeleder. About two years ago he was informed that a lady of his acquaintance, suffering from an ovarian cyst, who had been much relieved in Dresden by acupuncture (? galvanopuncture), had been ultimately cured in Vienna by the same treatment. Since that time he has tried it in three cases:

1. A young lady, aged eighteen, who had a soft fluctuating ovarian tumor, originating on the left side and extending three centimeters above the umbilicus, was subjected to galvanopuncture. In four months the diameter of the abdomen, two inches below the umbilicus, was reduced from ninety-six centimeters to ninety-two centimeters, and in two months more the cure was completed.

2. A lady twenty-four years old, and the mother of two children, had a tumor in the lower part of the abdomen, on the left side, as large as the head of a child two years of age. When she had been under treatment two months the patient was cured, the remains of the cyst being hard, and of the size of a small apple.

3. A woman forty years of age, with a tumor reaching up to the umbilicus, had so far recovered at the end of six weeks of the treatment that its continuance was considered unnecessary.

No unpleasant consequences occurred in either of these cases, and none of the cysts have refilled. The author considers that the action is the same as that which occurs when the poles of a battery are placed in an albuminous fluid, viz: clotting and thickening at the positive pole, and liquefaction at the negative. He considers the method equally applicable to multilocular and unilocular cysts. He does not give an exact account of his method of procedure, but each sitting was of short duration. He anticipates equally favorable results in the treatment of hydatid cysts on this plan.—*Med. and Sur. Rep.*

*A New Antiseptic Dressing.*—Boracic acid in fine powder, one part; white wax, one part; paraffin, two parts; almond oil, two parts. The ingredients, after being mixed by melting the wax and paraffin, are stirred in a warm mortar till the mass thickens, and then set aside to cool, after which the fine substance is reduced in a cold mortar, in successive portions, to a uniform soft ointment. This is spread thin on a fine rag, and when the almond oil leaves, as it soon does through the capillary attraction of the porous external dressing, a smooth, firm layer remains, which can be separated from the skin without leaving any greasy substance adhering, and does not at all confine the discharge which, whle freely shed, is perpetually supplied with a sufficient quantity of boracic acid to insure absence of putrefaction, while not preventing cicatrization. Another, and, it is thought by Dr. Henry, a better application in cases of this nature, is an ointment composed like the one above described, except that instead of one part of boracic acid, it contains half the quantity of salicylic acid, which, while possessing very remarkable antiseptic powers, is even less irritating than boracid acid.—*Advance.*

*Mistura Glycyrrhizæ Composita.*—In place of the usual method, which consists in rubbing together with water, licorice, sugar and gum arabic, I propose using the officinal simple syrup and mucilage of gum arabic and a solution of the extract of licorice (made by dissolving the extract in water), of such a strength that f oz. ii shall represent oz. i of the extract.

The formula is as follows:

R. Solution of extract of licorice.........f oz. i.
Syrup......................................f dr. v.
Mucilage of gum arabic.....................f dr. xi.
Water, a sufficient quantity to make...f dr. xiiss.
Camphorated tincture of opium.......f oz. ii.
Wine of antimony...........................f oz. i.
Spirits of nitrous ether...................f oz. ss

Mix.—*W. E. Bibby in the Journal of Pharmacy.*

*Oleum Ovorum, or, Oil of Eggs*, is prepared as follows: A suitable number of eggs are broken into a tinned vessel, and heated on the water-bath under constant stirring, until the mass has assumed the consistence of a soft ointment, and a small portion pressed between the fingers shows traces of oil. The whole mass is enclosed in a linen bag, placed between two boards, immersed in hot water, and forcibly pressed. The resulting oil is filtered and kept in small, well-closed vessels. Two eggs yield four gm. of a thick oil, free from rancidity. The same may also be prepared by heating eggs in a porcelain vessel on the water-bath until they are reduced to a dry, pulverulent mass, which is placed into a displacement apparatus and exhausted with carbon disulphide. The latter is evaporated or distilled off, and the oil remains behind.—*New Remedies.*

*Boracic Acid as an Ordinary Dressing for Wounds.*—Dr. Leonard Cane extols (*Lancet,* May 20th, 1876,) boracic acid as a dressing for wounds. "Its advantages, he states, are: 1st. It is an antiseptic which does not irritate and inflame, and so allows the natural processes of healing to go on without much interruption.

"2d, It is exceedingly simple in its application, and can be used apart from all the details required by a thoroughly antiseptic method.

"3d, It can be used in the shape of the lint, lotion, cotton-wool, etc., in combination with most other methods of treatment.

"4th. Its cost is trifling; and though this is of secondary importance, it is a feature of the treatment which will recommend its employments in workhouse infirmaries and in dispensary and parish practicc."—*American Medical Journal of Medical Seience.*

*Offensive perspiration* of the feet is often quite as difficult to cure as it is troublesome to endure. Although the perspiration of the general surface of the body may be in some cases offensive, that of the feet may be made worse by decomposition of sebaceous matter, and very much can be accomplished by preventing this by frequent changing of the socks and shoes. If the case is not an aggrevated one, bathing the feet daily with alum-water may lessen the secretions. Hebra and Martin recommend that each foot be strapped for twelve hours at a time with diachylon plaster. The most recent remedy is the use of salicylic acid mixed with powdered soapstone and dusted into the socks. This is supposed to prevent decomposition of the sebaceous matter, and consequent development of bad-smelling acids.—*New Remedies.*

*Salicylic acid* can be very conveniently given in the doses of ten and fifteen grains by enveloping the powder in wafers or the "cachets de pain." It has been administered in this way in fifteen-grain doses for two or three weeks without causing any disturbance whatever, and at the same time producing very notable reduction of temperature.—*New Remedies.*

*Cholera Infantum.*—Egg-water, made by dissolving the whites from four eggs in a pint of iced water, to which a teaspoonful bicarbonate of soda has been added, is, in my opinion, one of the very best articles of diet in cholera infantum. By the use of the drink I have seen patients rescued from imminent danger of collapse. It is taken with evidity by very young children, and is very seldom ejected, is readily digested, the albumen passing into the circulation and replacing that element of the blood exuded in the watery evacuations. In some cases I have administered, with the best results, the white of an egg, beaten well with a spoon, to which a lump of ice had been added.

*Crayons of Tannin.*—In the *Annales de Gynecologic*, May, we find the following formula for the preparation of crayons of tannin: To fifteen grains and a half tannin add a drop and a half of glycerine, and make a crayon nearly four inches in length. Crayons thus made will keep their forms for months; they may be lengthened or shortened as required, after simply warming them in the fingers, and yet are sufficiently firm tc be passed into the uterine cavity without breaking them. In consequence of their ductility, they may be lengthened so as to make them into astringent bougies, and then introduced into the urethra will be an efficient substitute for tannin injections.

*Leroy's Purgative Elixir*—

| | |
|---|---|
| Scammony | 2 oz. |
| Turpeth root | 1 oz. |
| Jalap | 8 oz. |
| Brandy, or diluted alcohol | 12 p. |

Digest twelve hours at a heat of about one hundred and twenty-two degrees F., strain and add the following syrup:

| | |
|---|---|
| Senna | 8 oz. |
| Boiling water | 2 p. |

Infuse, press out the liquid, and add:

| | |
|---|---|
| Brown sugar | 2½ pounds. |

Mix together, and strain or filter. Dose, one to four tablespoonfuls.

*Pruritus in Pregnancy.*—In answer to the inquiry of Dr. G. L. C., of South Carolina, concerning "Pruritus in Pregnancy," I suggest he tries—

R. Sodæ borax............................ounce ss.
Morph. sulph.,..........................grains vj.
Aquæ rosæ distil.,..................... ounces viij. M.

Apply three or four times daily with a bit of sponge, first washing the part with tepid water and soap, and dry them before applying the lotion.

I have used the above (taken from Colombat, by Meigs, page 276) in a number of cases without a single failure.—Samuel E. Wills—*Med. and Surg. Rep.*

*Enuresis.*—Dr. Monette (*American Practitioner*) says: I tried the tincture of cantharides combined with chloral. The combination fulfilled the indications in re-establishing the tonicity of the vesical sphincter, as well as modifying the excessive irritability of the muscular coat of the bladder. The tincture of cantharides must not be used in maximum doses, or till strangury is produced, the age and strength of the child being taken into consideration. In enuresis I have used cantharides alone to verify its efficacy, and have also used it to palliate the strangury often present in cystitis, also in cases where enuresis complicated cystitis.

*Tapping the Gall Bladder.*—Dr. E. L. Dixon relates (*The Practitioner*, April, 1876,) a case in which the gall bladder was tapped five times, not merely with impunity but with great relief to the patient, and a total of 87½ oz. of liquid withdrawn by the aspirator. The patient was jaundiced, her gall-bladder enormously distended, the common bile duct occluded by malignant deposit. The life of the patient seemed to have been protracted by the operation, but, as might have been expected, a fatal result was not prevented.—*American Medical Journal of Medical Science.*

*Resolvent Ointment.*—The following ointment is employed at the Hotel-Dieu with great success, by Dr. Noel Gueneau de Mussy: Camphor one, chlorhydrate of ammonia four, and lard thirty parts.

*Stove Polish as an Irritant.*—A correspondent suggests that a fertile source of catarrhal and bronchial irritation may be found in the use of stove polish. This being of metalic substance and of a drying nature, is expelled by heat, and mingles with the air of the room, and in this state of minute division finds its way into the respiratory tract, adhering to the first moist substance it meets. Hence the pharynx and Schneiderian membrane suffer rather than the lungs. He thinks follicular pharyngitis is often maintained by these exhalations.—*Med. and Surg. Rep.*

*Convulsions Arrested by the Sinistro-Lateral Posture.*—Dr. F. J. Brown has seen two cases of convulsions arrested by turning the patient over upon the left side. One patient, a man with Bright's disease, had uræmic convulsions, which ceased instantly after he had been turned upon the left side. Another man, who had been seized with unilateral convulsions, was relieved in fifteen seconds after turning upon the left side. Dr. Brown's theory is that this posture is in some way beneficial by favoring the heart's action. *The Practitioner, April,* 1876.

*Hair Restorer*—

| | |
|---|---|
| Bay rum | 2 oz. |
| Glycerine | 2 oz. |
| Tincture of cantharides | 1 dr. |
| Oil of bergamot | 30 drops. |
| Sulphate of quinia | 10 gr. |
| Water | 4 oz. |

Mix. Use every morning for dressing the hair in place of hair-oil or pomatum.

*Aneurism involving the Innominate, First Part of the Subclavian as well as Part of the Arch of the Aorta.*—A patient of Mr. Spence, who had suffered from this trouble, was exhibited (April 5) to the Medico-Chirurgical Society of Edinburg. Before anything in the way of operation was attempted, rest was tried, and half-drachm doses of iodide of potash were given four times a day. This treatment proved successful, so that there was new consolidation and absence of all pulsation.—*Ed. Med. Journ.*, May, 1876.

*Imperfect Mastication as a Cause of Diarrhœa.*—Dr. A. W. Edis calls attention (*The Practitioner*, April, 1876,) to what he justly considers a frequent cause of diarrhœa, viz., deficient mastication from defective or decayed teeth. It is also most certainly a very frequent cause of dyspepsia in various forms, and the only mode of relief for these ailments is by having adjusted properly in the mouth artificial teeth to assist in mastication.—*American Medical Journal.*

*Cure for Warts.*—Lisfranc immerses the parts on which the warts are developed in a strong solution of black soap. This causes a slight cauterization of the surface of the wart. The loosened tissue is to be removed and the application repeated every day till the cure is complete. Oil of vitriol should never be used for this purpose; it is very irritating, and inflames the warts instead of curing them.—*Trib. Med.*, No. 316, 1874.

*Cure for Corns.*—A correspondent of the *Scientific American* writes as follows, to one who asks for a remedy for corns: Bind raw cotton on your corn at night before going to bed, and then saturate the cotton with spirits turpentine. It will remove the most obstinate corn, either hard or soft, in four or five applications. The skin will be apt to peel off the toe, but this is rather an advantage, as it helps to remove the corn.

*Harmless Cosmetic Powders.*—The apothecaries of Copenhagen have agreed on the substitution of harmless compounds for the numerous poisonous face-powders now commonly used. In avoirdupois weight, the proportions of the ingredients will be about as follows: For white powder, oxide of zinc, 1 oz.; wheat starch, 9 oz.; oil of rose, three drops; for red powder, carmine, 1 oz.; carbonate of magnesia, 4 oz.

*Chromic Acid for Warts.*—Three or four applications of this acid will cause the disappearance of warts, however large, hard or dense they may be. The application gives rise to neither pain, suppuration nor cicatrices, the sole inconvenience being the production of a dark brown color.—*Union. Med. Times and Gazette.*

*To Remove Stains Produced by the Nitrate of Silver.*—A few centigrammes of metallic iodine are placed in a saucer, to which a few drops of ammonia are added; then by means of a brush, or simply with the finger, the solution is applied to the stains. However old or extensive the stains may be, they disappear immediately. It is important to destroy the mixture afterward, since it is nothing more or less than the iodide of nitrogen, which when dry is an explosive body. This procedure is decidedly superior and more prompt in its action than the application of a solution of iodine or of the cyanide of potassium, without possessing the disadvantages of the latter.—(*L'Abeille Medicale.—Le Bordeaux Medicale.*)

*Fatal Poisoning by Alcohol.*—I. J. Radcliffe, M.D., described in the *Medical Times*, of June 24, the case of a child, aged four years and five months, who was fatally poisoned by drinking about seven ounces of whisky, of inferior quality. The whisky was taken in the morning, fasting; was rapidly absorbed, and in fifteen minutes the child was utterly insensible. In spite of all efforts, he died in about twelve hours, the immediate cause of death appearing to be congestion of the brain and lungs. The pupils were widely dilated, respiration was irregular, the pulse was quick and irregular, and about three hours before death took place, convulsive movements occurred.—*New Rem.*

*Gum Arabic Mucilage.*—This preparation, when made according to the pharmacopœia, does not keep very well in warm weather, and Messrs. Archer & Co., in the *American Journal of Pharmacy*, recommend that it be made with tolu water. The mucilage thus prepared has a faint odor of tolu, and is, in appearance, similar to the officinal. The tolu water is prepared by rubbing two fluid drachms of a saturated tincthre of tolu with four drachms of magnesium carbonate, and two pints of water and filtering. The tolu is supposed to prevent the mucilage from changing, upon the same principle that benzoin obviates rancidity in fats.—*New Remedies.*

*Oil of Eggs for Sore Nipples, Chaps, etc.*—Heat yolk of egg until it becomes thoroughly dry; press it, and digest in boiling alcohol; filter while hot, and distil off the spirit.—*New Rem.*

*Painful Menstruation.*—Dr. Baker, of Norristown, has found the following formula, given a week or ten days before the menstrual period, to yield almost sure relief in painful menstruation:

R. Pil. ferri carbonat........................dr. iij.
Ext. conii mac..............................dr. ijss.
Ol. cinnamon..............................min. xxx.
Syr. tolutani................................oz, ij.
Syr. simplici.
Aquæ,........ .........*aa*................oz. vij. M.

Sig. A tablespoonful four times a day.

*Lime Water in Infantile Eczema.*—A writer in the *Bulletin de Therapeutique* recommends lime water in eczema of the head and impetigo of the face in children, especially chronic cases, which have resisted other treatment, and states that a marked improvement is noticeable after using it for eight days. It is to be taken in quantities varying up to half a pint, according to the age of the patient, and to dust the part with carbonate of magnesia; but the latter is only necessary when the secretion is very irritant.—*Med. & Surg. Rep.*

*Mode of Administering Creosote.*—As creosote is now frequently employed in the treatment of typhoid fever, and is exceedingly distasteful to some patients, it may be worth while to mention here a formula which, in a great measure, covers its flavor, and is easily prepared: Creosote, 3 drops; essence of lemon, 2 drops; orange flower water, 1 ounce; spring water, 3 ounces. A spoonful to be taken at frequent intervals throughout the day.—*Canada Medical Journal.*—*Virginia Clinical Record.*

*Clorate of Potassium in Chronic Gastro-Intestinal Catarrh.*—S. C. Dumm, M.D., used chlorate of pottassium in a case of chronic gastro-intestinal catarrh, attended with inflammation of the bile duct, with excellent results Ten grains were given every three hours, dissolved in water. After the use of the remedy for one day, pain in the stomach ceased, the appetite improved, vomiting stopped, and the patient recovered her heath quickly.—*New Remedies.*

*Pure English.*—" A doctor was lately summoned to a cottage at Harwood, in Teasdale, and found a boy in need of his services. ' Put out your tongue,' said the doctor. The boy started like an owl. ' My good boy,' requested the medical man, ' let me see your tongue.' ' Talk English, doctor,' said the mother; and then turning to her son, she said: ' Hoppen thy gobbler, and put out thy lolliker.' The boy rolled out his tongue in a moment."—*English Paper.*

*Absorptive Power of Milk.*—Attention has been called in the daily papers, to a practice prevalent in some parts of the country, which appears to illustrate the power possessed by milk of absorbing atmospheric impurities. It is that of placing a saucer of new milk in a larder, to preserve meat or game from taint. It is said that not only does it answer that purpose, but that the milk in a few hours becomes so bad that no animal will touch it.—*London Lancet.*

*Foot Powder.*—It is stated that salicylic acid removes the odor of sweat from the feet, without preventing the sweating; its action being to prevent the formation of butyric, valerianic and other acids of the same family, which injure the feet. M. Paulcke, therefore, prepares with salicylic acid, soap, talc and starch, a powder for the feet, which while rendering them firm, is said to induce an agreeable softness, and removes all unpleasant smell.—*New Remedies.*

*Erasive Soap to Remove Grease and Stains from Clothing.*—Two pounds of good Castile soap, half a pound of carbonate of potash, dissolved in half a pint of hot water. Cut the soap in thin slices, boil the soap with the potash until it is thick enough to mould in cakes; also add alcohol half an ounce, camphor half an ounce, hartshorn half an ounce; color with half an ounce of pulverized charcoal.—*Scientific American.*

*Cod-Liver Oil.*—M. Guichard states that for many persons cod-liver oil can be sufficiently disguised by breaking up sardines in it. The person should first be taught to like sardines, and then it is but a short step to cod-liver oil with sardines crumbled in it.—*New Remedies.*

*A New Adhesive Plaster*—A mixture of twenty parts of mucilage and one part of glycerine constitutes an excellent shining and supple plaster, far cheaper than the rosin and diachylon, and lasting more than a year without deterioration. Three or four layers of the mixture require to be spread over each other on the linen or other stuff, allowing sufficient intervals for the successive layers to dry.—*Med. & Surg. Rep.*

*Hypodermic Injection of Cold Water for the Relief of Pain.*—Dr. S. Henry Dessau, of this city, reports in the *New York Medical Journal*, of June, seven cases in which he followed the suggestion of Dr. Lelut, (*L'Union Medicale*), in the use of cold water, introduced hypodermically, for the relief of pain of a rheumatic character. In all the cases the operation worked well.—*New Remedies.*

*Remedy for Sick Headache.*—Dr. R. H. McKay, of Tennessee, contributes to the *Med. and Surg. Reporter* the following formula, which he has found of great value in the treatment of sick headache: Granulated muriane of ammonia, one teaspoonful; acetate of morphia, one grain; water, half a pound. Dose for an adult, two teaspoonfuls every ten minutes (precisely) until relief is obtained.

*Camphorated Ether in Erysipelas.*—According to Cavazzani, a preparation consisting of camphor and tannin, of each one part, and ether eight parts, is an excellent application, even in cases of phlegmonous erysipelas. It is also very efficacious in burns of the first and second degrees; the heat disappears rapidly, and blisters do not form. It should be applied every three hours.

*Dysentery.*—Some cases of dysentery exist at the present time in the medical wards. The treatment that proves most efficacious consists in the administration of powders containing one-fourth of a grain of opium and ten grains of tannin.—*Roosevelt Hospital, New York Medical Journal.*

Sir James Paget says, the best wash for hardening the skin, to prevent bed sores, is one part of sweet spirits of nitre to three parts of water.—*New Remedies.*

*Catarrh Snuff.*—Dr. E. C. Mann, of New York, recommends the usc of a snuff composed of equal parts of finely pulverized camphor and white powdered sugar as an adjuvant to the various injections and sprays employed in the treatment of nasal catarrh.—*New York Medical Journal.*

*Jalap Biscuit*—Mr. Tambureau, of Algeria, proposes as a convenient purgative a biscuit containing jalap, resin, sugar, eggs and flour, and flavored by vanilla. The biscuits thus produced are said to have a presentable appearance and agreeable taste.—*New Remedies.*

*Dentifrices.*—M. Paulcke, a pharmacien at Leipzig, prepares as a dentifrice a powder in which salicylic acid is incorporated; also an elixir "dentifrice," from a solution of the acid aromatized with oil of wintergreen.—*New Remedies.*

A Paris medical journal says that a resolvent ointment employed at the *Hotel Dieu* with great success, consists of one part of camphor, four parts of chloride of ammonium, and thirty of lard.—*Boston Jour. Chem.*

In the ireatment of burns in the Charity Hospital, New York, when of a superficial character, a preparation consisting of two parts of collodion and one of olive oil, has been found to be very efficacious.—*Boston Jour. Chem.*

Inspector-General H. Franklin, C.B., who died at Folkestone, England, on the 2d of August, aged eighty-two years, was a surgeon in the British army at the battle of Plattsburg.—*Medical Record.*

